# New technology: clinical efficacy of intracapsular decompression and supported hollow screw treatment for Garden IV femoral neck fractures

**DOI:** 10.1186/s40001-024-02161-3

**Published:** 2024-11-27

**Authors:** Zhaofeng Jia, Tinghui Xiao, Yimiao Lin, Wenjun Huang, Peizhi Deng, Jiandong Lin, Shiyuan Lin, Jiwu Cheng, Fengting Cui, Xinjia Hu, Xiaoming Zhang

**Affiliations:** Department of Traumatic Orthopedics and Institute of Orthopedic Research, Shenzhen People’s Hospital, The Second Clinical Medical College of Jinan University and the First Affiliated Hospital of Southern University of Science and Technology, Shenzhen, 518035 Guangdong China

**Keywords:** Intracapsular decompression and supported hollow screw, Femoral neck fracture, Garden IV, Minimally invasive percutaneous treatment

## Abstract

**Background:**

The management of Garden IV femoral neck fractures presents a formidable challenge. This study aimed to assess the safety and efficacy of a novel technological approach for treating Garden IV femoral neck fractures, involving intracapsular decompression and the utilization of supported hollow screws.

**Methods:**

Between October 2018 and October 2021, a cohort of 46 patients, comprising 25 males and 21 females, was admitted for Garden IV femoral neck fractures. The surgical intervention employed a novel methodology, involving closed reduction of femoral neck fractures on a traction bed and percutaneous implantation of decompression support hollow screw internal fixation in the capsule. Operative parameters such as intraoperative bleeding volume, incision length, and fluoroscopy frequency were recorded. Postoperative angiography was performed to evaluate the blood supply to the femoral neck and femoral head, while long-term follow-up records documented femoral neck fracture healing and patient functional recovery.

**Results:**

The closed reduction and minimally invasive implantation of decompression support screws in the femoral neck capsule proved effective in successfully fixing Garden IV femoral neck fractures. The screw placement time was 20–45 min, with an average of 25.2 min. The number of fluoroscopy was 6–14, with an average of 7. The postoperative follow-up was 20–25 months, with an average duration of 22 months. All patients exhibited uneventful single-stage wound healing, devoid of complications such as nerve, blood vessel, or important tissue structure injuries. While femoral head necrosis occurred in one case and femoral neck shortening in another, all femoral neck fractures exhibited healing. Post-surgery, patients experienced rapid recovery, enabling early functional exercise with satisfactory functional restoration.

**Conclusion:**

The innovative technology, employing femoral neck capsule decompression support hollow screws, not only offers structural support, but also facilitates capsule decompression post-femoral neck fracture surgery. This approach promotes the restoration of blood flow to the femoral head and neck, accelerates fracture healing, diminishes the likelihood of femoral head necrosis, and enhances the overall therapeutic outcome for Garden IV femoral neck fractures. The merits of this novel technique warrant its widespread adoption and application.

## Introduction

Femoral neck fracture is a prevalent orthopedic affliction, constituting approximately 50% of hip fractures [1]. The Garden IV femoral neck fracture, characterized by complete displacement, typically arises from high-energy injury [2]. Given the evident displacement, surgical intervention is commonly imperative [3]. However, owing to the anatomical and biomechanical characteristics of the femoral neck, high-energy injury frequently results in an unstable fracture configuration in Garden IV femoral neck fractures, characterized by incomplete femoral calar, vertical fracture section, and vulnerability to posterior lower cortex crushing. Therefore, the prevalence of secondary surgeries, bone nonunion, femoral head necrosis, and internal fixation failures is elevated, rendering its treatment is extremely challenging [4].

Studies have suggested that the occurrence of fracture nonunion and femoral head necrosis subsequent to Garden IV femoral neck fractures can be attributed, in part, to arterial insufficiency and impeded venous return caused by heightened capsular pressure post-fracture. This instigates a prolonged state of ischemia and hypoxia at the fracture site and femoral head, thereby impeding fracture healing and femoral head nutrition [5]. Conversely, the complete displacement of the fracture end in Garden IV femoral neck fractures, coupled with femoral neck shear forces, engenders considerable instability. If internal fixation fails to furnish adequate shear force and anti-rotation capabilities, the risk of fixation failure escalates, culminating in fracture nonunion and potential femoral head necrosis [6].

Currently employed clinical fixation methods for Garden IV femoral neck fractures encompass closed reduction, percutaneous three hollow screws internal fixation, proximal femoral plate fixation, dynamic hip screws, dynamic cross screws, and other surgical modalities [7]. Closed reduction percutaneous hollow nail fixation has garnered increasing favor among clinicians due to its advantages of reduced trauma, straightforward execution, and cost-effectiveness for patients. While dynamic hip screws can offer continuous compression and fixation at the fracture site, their substantial impact on bone integrity and blood supply, coupled with the inability to achieve minimally invasive fixation, extends patient recovery time [8, 9]. The prevalent use of three screws (3 cannulated screws, 3CS) in treating femoral neck fractures poses challenges such as screw withdrawal, femoral neck shortening, and coxa vara, leading to complications that are detrimental to hip function recovery [10]. While some recent reports highlight favorable clinical outcomes following locking plate reduction, this procedure necessitates open reduction, further disrupting blood flow at the fracture site, yielding less satisfactory results [11, 12]. Presently, it is widely accepted that the key of treating Garden IV femoral neck fractures lies in achieving precise reduction, capsular decompression, and robust internal fixation [13, 14].

**Fig. 1:**
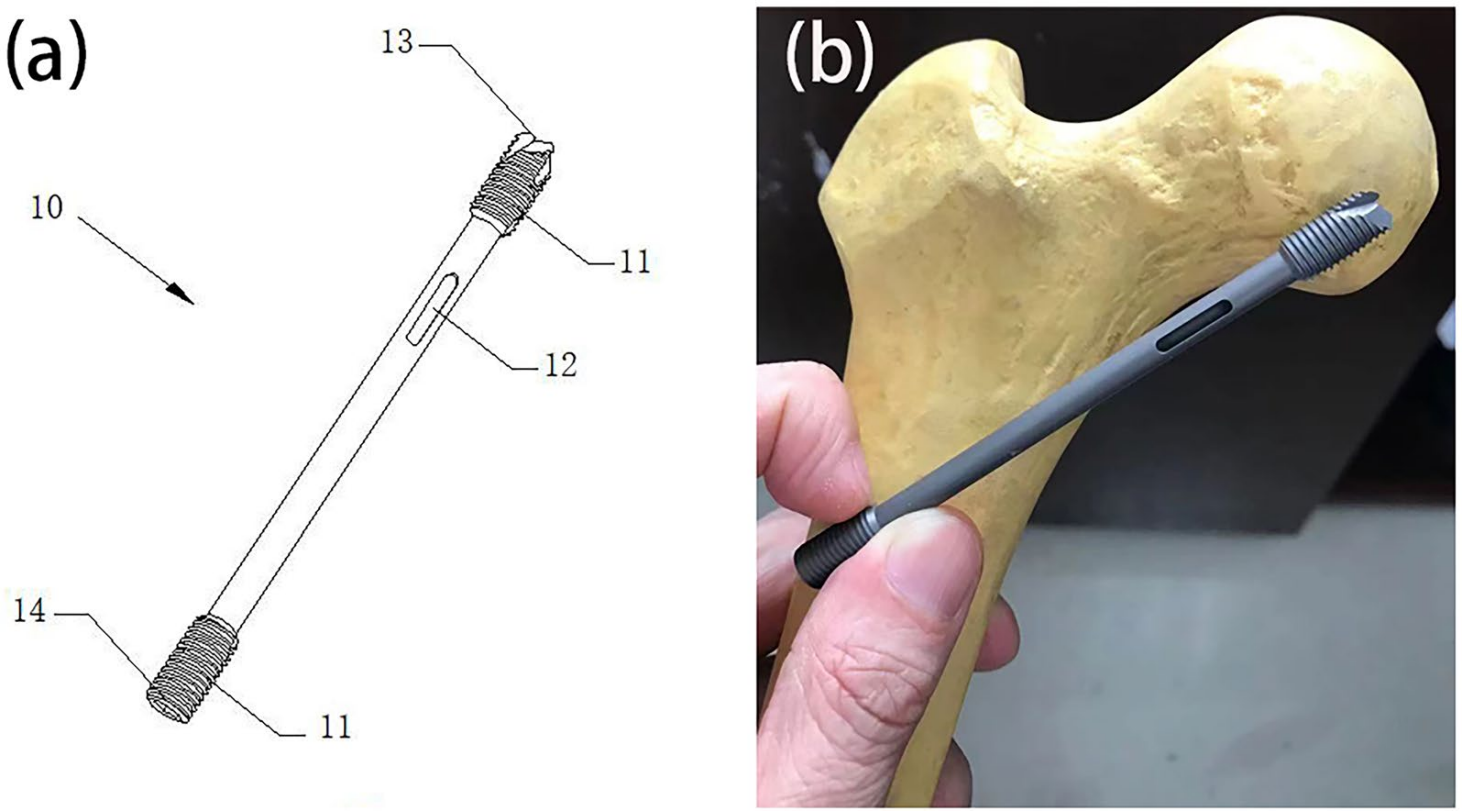
Design and implantation of hollow screws for decompression support in femoral neck capsule. **a** Hollow screw design for femoral neck capsule decompression support. **b** Schematic representation of hollow screws implanted for decompression support in the femoral neck capsule

The resolution of intracapsular decompression and the provision of effective mechanical support following a femoral neck fracture are paramount in the management of Garden IV femoral neck fractures. Therefore, our research team has introduced an innovative intracapsular decompression support device in the form of a hollow screw (Fig. 1). Additionally, a set of three hollow screws has been devised for combined application, exerting a synergistic effect of continuous capsular decompression and medial static stabilization support. This approach furnishes robust support and fixation to the femoral neck while alleviating capsular pressure at the fracture site. Subsequent to clinical follow-up observations, the novel technique has demonstrated favorable outcomes in the treatment of Garden IV femoral neck fractures.

A retrospective study involving 46 patients with Garden IV femoral neck fractures admitted to our department from October 2018 to October 2021 was conducted to evaluate the clinical efficacy of closed reduction and transcutaneous decompression-supported hollow screw and semi-threaded hollow nail fixation. The following report presents the findings of this retrospective study.

## Materials and methods

### Ethics statement

Approval for this retrospective study was obtained from the Ethics Committee of Shenzhen People's Hospital at Jinan University. All participants provided informed consent before participating in the study, ensuring adherence to ethical standards.

### Case inclusion and exclusion criteria

Inclusion criteria: (1) Clear history of trauma, age between 18 and 65 years. (2) Preceding normal hip joint function and the ability to walk without impairment. (3) Imaging diagnosis consistent with Garden IV femoral neck fracture.

Exclusion criteria: (1) Coexistence of other hip fractures or dislocations. (2) Presence of old or pathological fractures. (3) Concurrent serious medical conditions rendering the patient unfit for surgery. (4) Prolonged usage of high doses of hormones or excessive alcohol consumption.

### General information

**Fig. 2:**
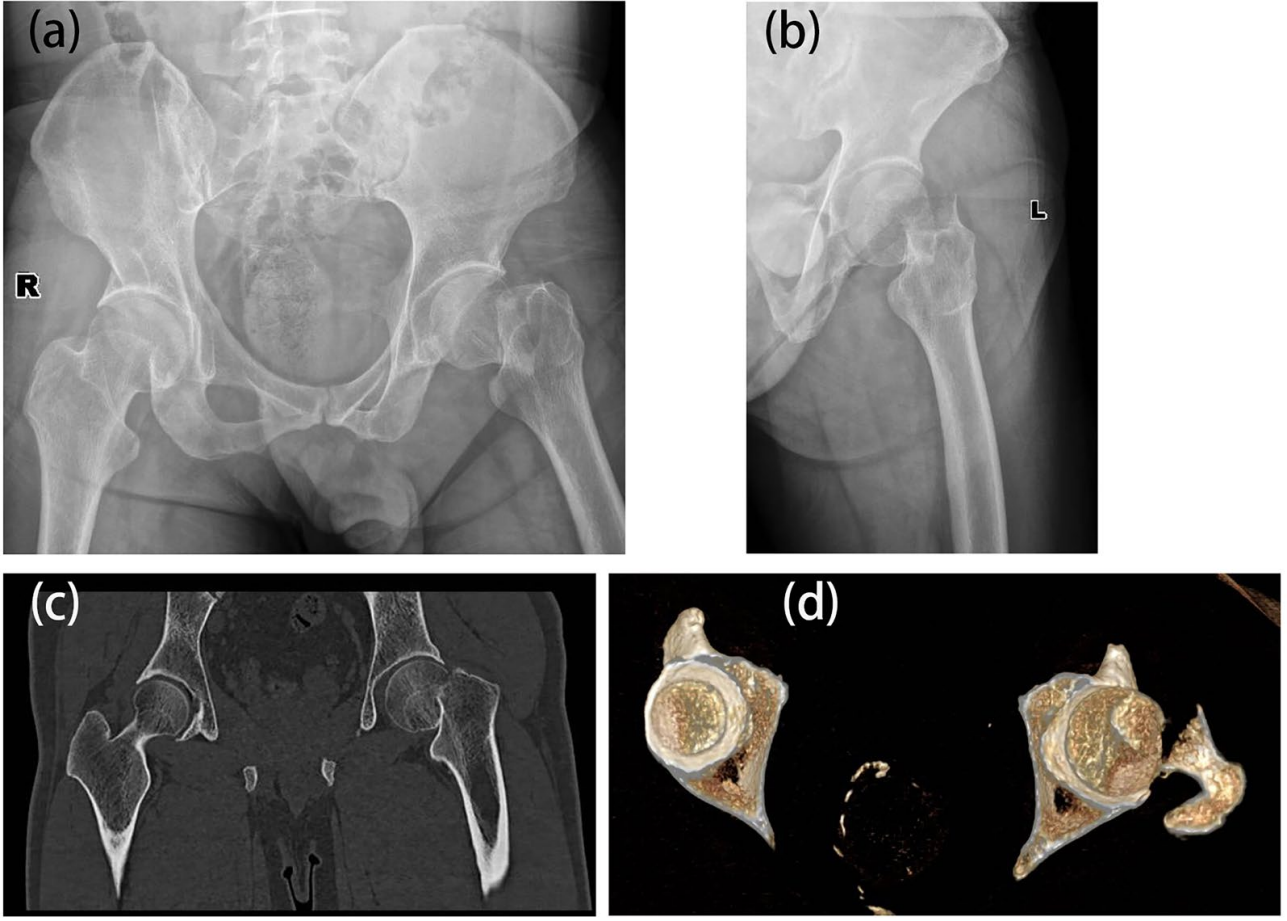
Preoperative X-ray and CT scan showing left femoral neck fracture (Garden IV type): **a** hip joint in an upright position; **b** left hip joint lateral position; c-d preoperative CT revealing complete displacement of the left femoral neck fracture

A cohort of 46 patients, comprising 25 males and 21 females, diagnosed with Garden IV femoral neck fractures, was included in this study. The age distribution ranged from 33 to 65 years, with a mean age of 45.2 years. Comprehensive patient information, encompassing gender, age, cause of injury, Garden classification of the fracture, and preoperative waiting time, is presented in Table 1. Representative preoperative imaging data are illustrated in Fig. 2. Prior to the operation, all patients provided informed consent. The study received approval from the hospital's Medical Ethics Committee, and both patients and their families consented to and signed informed consent forms for participation in the study.

**Table 1:** General data statistics of patients

Summary of patients’ information	
Number of patients	46
Gender	Male 25 (54.4%)
	Female 21 (45.6%)
Age	38.4 ± 16.5 years (33–65)
Side	Right 27 (58.7%),
	Left 19 (41.3%)
Causes of injury	Sports injury 19 (41.3%)
	Traffic accident 15 (32.6%)
	Fall from height 12 (26.1%)
Classification	Garden IV (100%)
The time from injury to surgery	1–6 (1.5 ± 2.6) days

### Surgical methods

The patients were treated with either intubation general anesthesia or intraspinal anesthesia in the supine position, with closed reduction conducted under fluoroscopic guidance using an orthopedic traction table. The quality of intraoperative reduction was assessed based on the Garden line index, with Grade I reduction achieving 160° and 180°, respectively. Stage II reduction involved 155° forward and 180° lateral movement; Stage III reduction comprised either 155° forward or 180° lateral movement; Level IV reduction entailed 150° forward and 180° lateral movement [15].

**Fig. 3:**
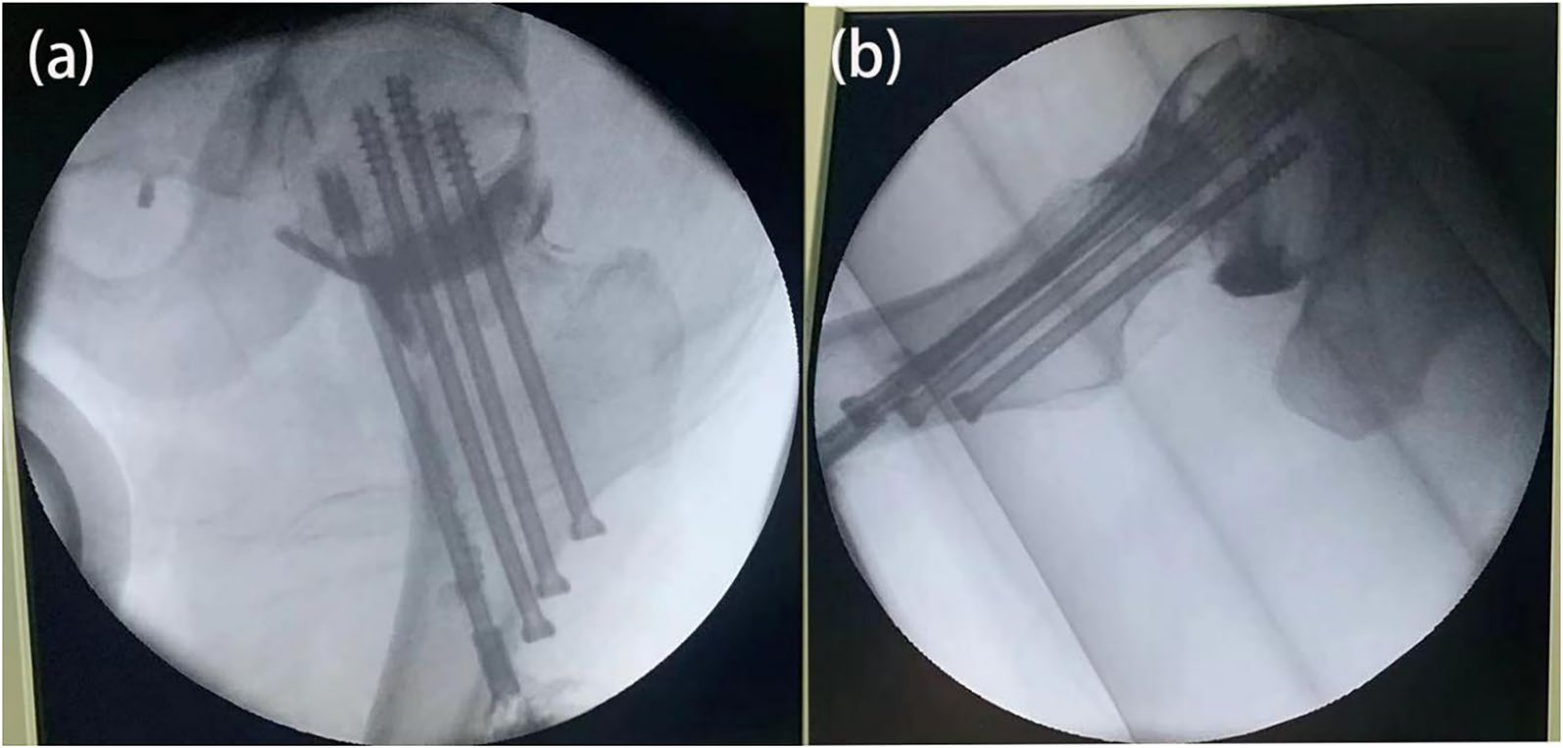
Intraoperative joint capsule angiography confirming that the decompression support screw in the femoral neck capsule is located inside the femoral neck joint capsule. **a** Left hip joint in an upright position; **b** left hip joint lateral position

Technical operation: Following successful reduction, a longitudinal incision of approximately 3–4 cm below the affected side trochanter was made. This incision was layered to access the proximal lateral cortex of the femur. Under anteroposterior and lateral fluoroscopic guidance, four guide needles were inserted from the lateral cortex of the femur, traversing the neck of the femur to reach the femoral head. These guide needles were arranged in a diamond-shaped pattern, with one proximal screw, two middle screws, and one distal screw (serving as the guide for the intracapsular decompression support hollow screw). The two middle guide pins were positioned anteriorly and posteriorly along the femoral neck, while the three guide pins closely adhered to the femoral neck wall. The guide pins for the intracapsular decompression support hollow screw were positioned beneath the femoral neck, passed through the femoral calar, and emerged beneath the femoral bone. The tips of all screws were situated approximately 5 mm below the surface of the femoral head cartilage, and then a hollow drill was employed to individually drill along the guide pins. The distal end of the drill bit traversed the fracture line and halted. The nail path's length was measured, and a 7.3-mm-diameter hollow nail was selected. The first three screws were sequentially threaded along the guide pins. The distal capsular decompression support hollow screw was positioned beneath the femoral neck, in the femoral neck capsule. The central through-hole of the adjustment screw was precisely located in the joint capsule, providing support beneath the femoral head. The screw tip was positioned around 5 mm below the cartilage surface of the femoral head. Intraarticular angiography was performed to confirm the screw's placement in the joint capsule (Fig. 3). Following satisfactory fracture reduction and proper screw placement, the operative wound was sutured in layers.

### Postoperative management

**Fig. 4:**
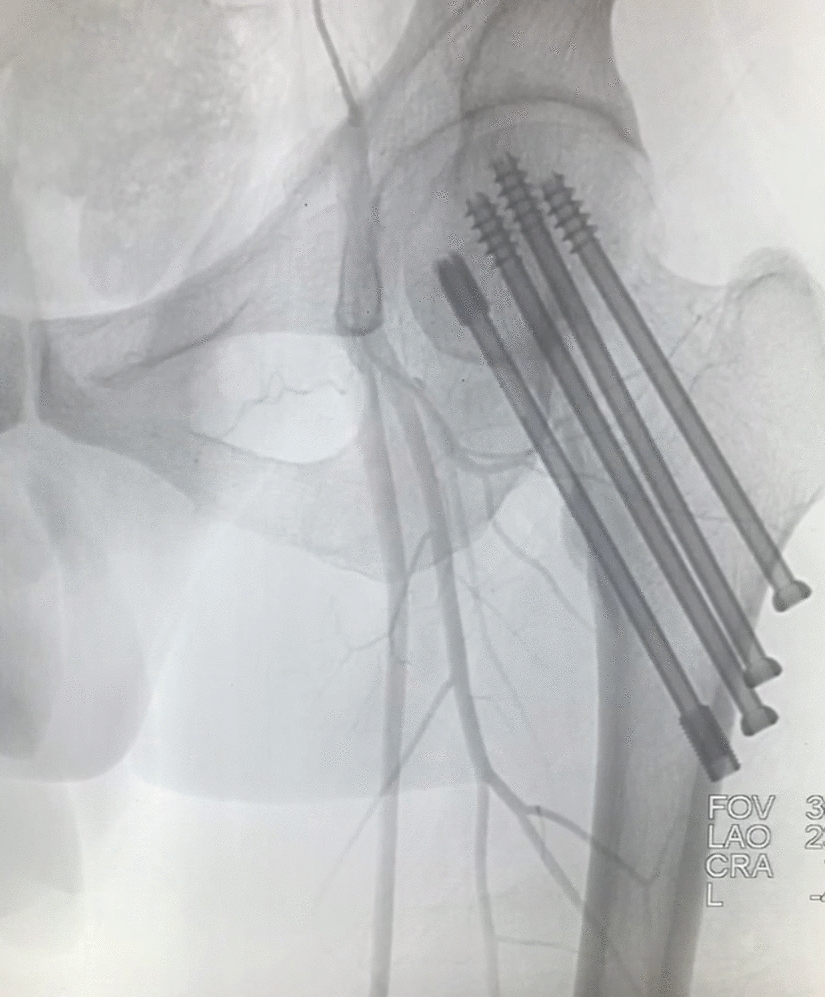
Postoperative angiography demonstrating the well-developed nutritional vessels in the femoral neck and femoral head, indicating preserved blood supply

**Fig. 5:**
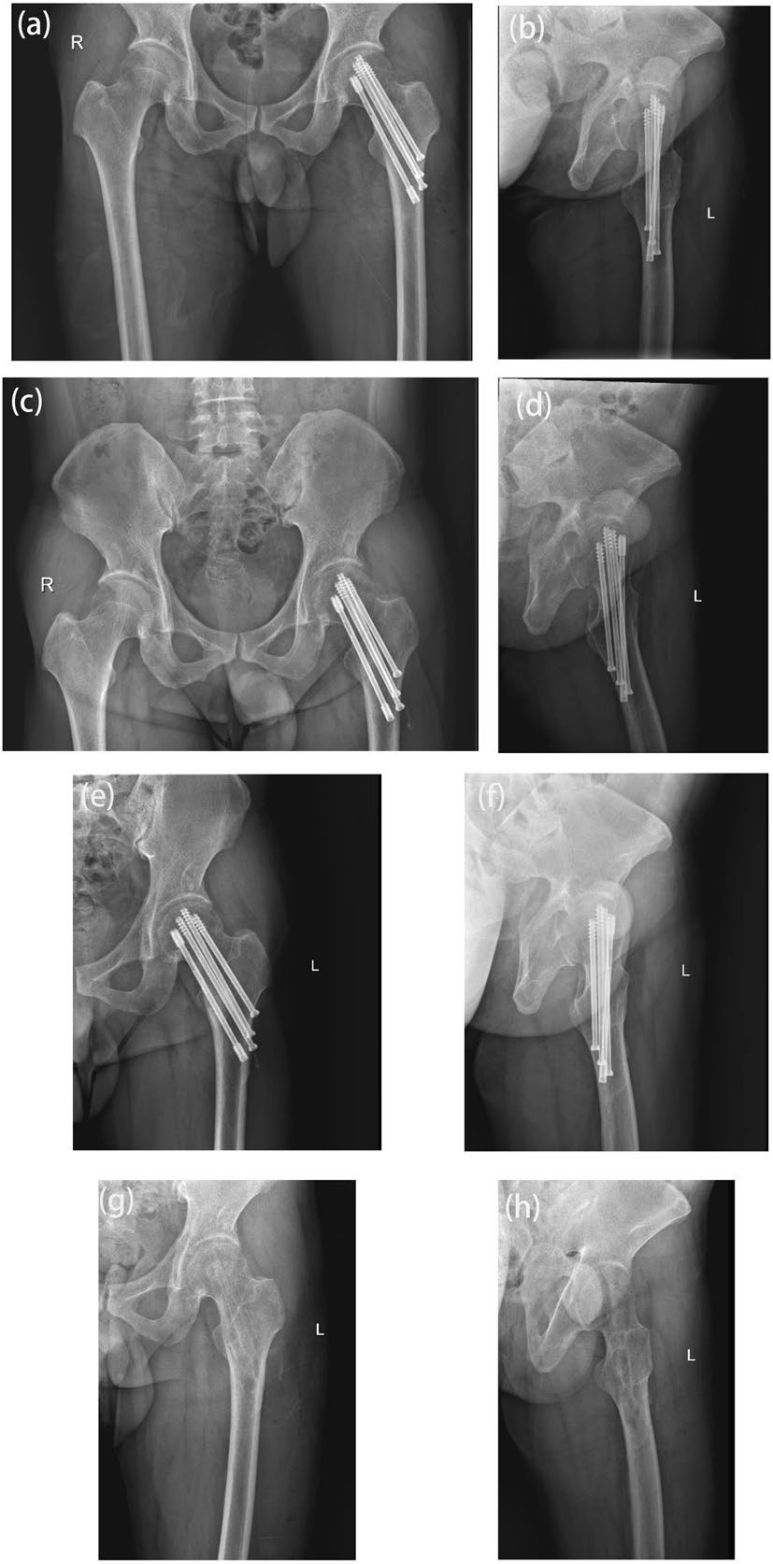
Postoperative follow-up X-ray confirming the successful healing of the left femoral neck fracture without femoral head necrosis. **a–b** Anteroposterior and lateral hip joint positions on the first day after surgery. **c–d** Anteroposterior and lateral hip joint views at 3 months post-surgery. **e–f** Anteroposterior and lateral hip joint positions one year after surgery. **g–h** Reassessment of hip joint anteroposterior and lateral positions after the removal of internal fixation

The lower extremity angiography of the patient was conducted in the intervention room on the second day post-surgery, confirming satisfactory blood circulation in the femoral neck fracture and femoral head (Fig. 4). All patients ceased antibiotic usage for 24 h, underwent a postoperative X-ray examination on the second day, initiated mobilization of the affected limbs with the aid of double crutches without weight-bearing on the third day post-surgery, and had stitches removed 2 weeks post-surgery. Subsequent X-ray assessments were routinely performed at outpatient clinics (Fig. 5), with patients progressively transitioning to full weight-bearing ambulation in alignment with fracture healing.

### Observation indicators

Perioperative parameters, time of last follow-up, duration until complete weight-bearing, Harris Hip Score, femoral head necrosis rate, fracture non-union rate, and incidence of femoral neck shortening were assessed. The evaluation of hip joint function employed the Harris Hip Joint Function Scale 16, which evaluates joint function, pain intensity, joint deformity, and range of motion. The total score ranges from 0 to 100, with higher scores denoting excellent (90–100 points), good (80–89 points), fair (70–79 points), and poor (< 70 points) outcomes, indicative of superior functionality [16].

## Results

**Fig. 6:**
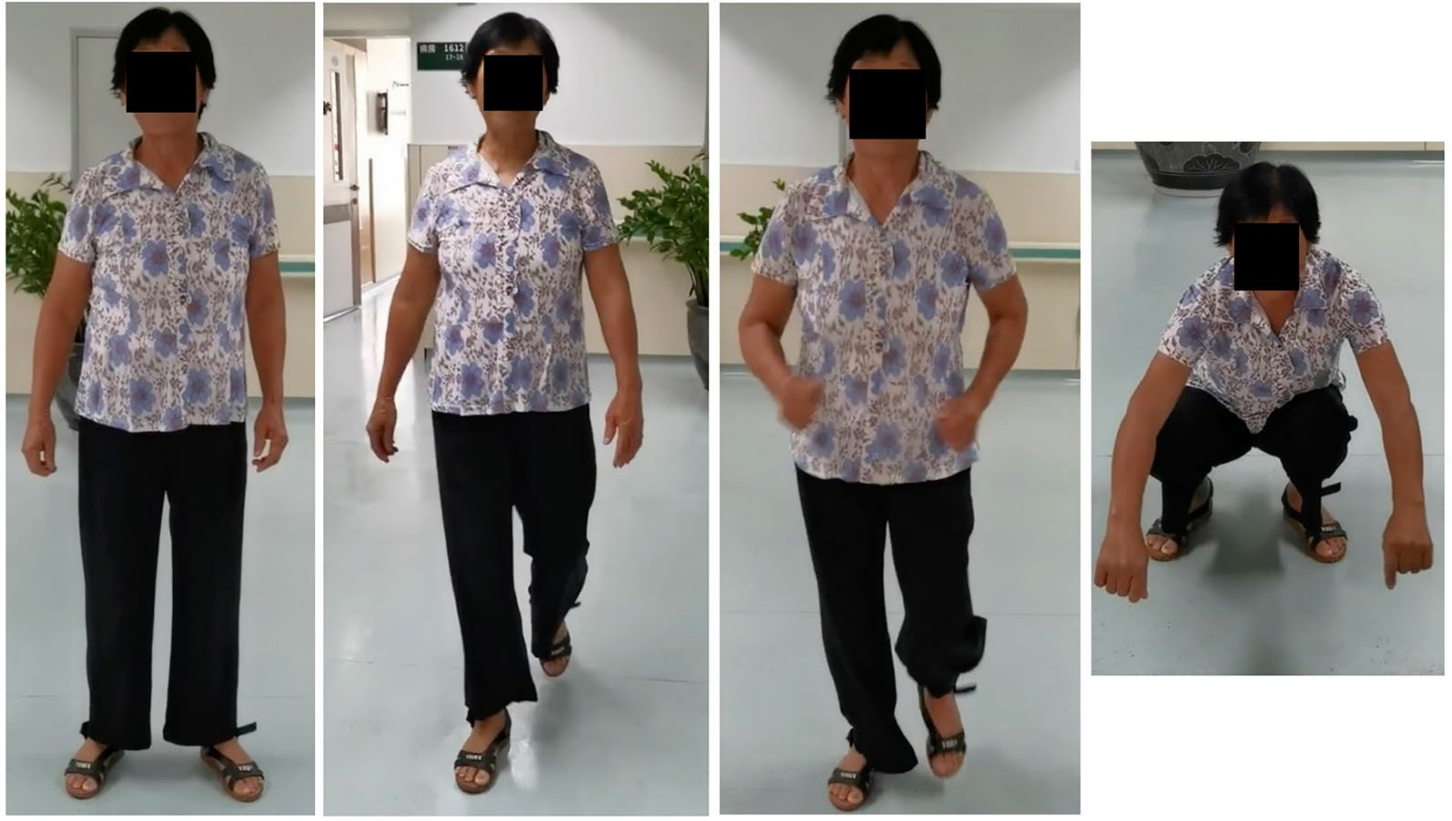
Satisfactory functional recovery observed in the patient, with normal standing, walking, running, and squatting activities

All patients underwent a follow-up period ranging from 20 to 25 months, with an average duration of 22 months. Successful closed reduction was achieved for all patients during the operation, and no complications such as neurovascular injury, fatalities, or postoperative incision infections were recorded. The operative time averaged 32.6 ± 12.4 min, with a blood loss of 55.6 ± 10.5 ml, incision length measuring 4.2 ± 1.6 cm, intraoperative fluoroscopy occurring 10.5 ± 3.5 times, and hospitalization lasting 8.3 ± 3.8 days. Postoperative follow-up revealed one case of femoral head necrosis, necessitating subsequent hip replacement surgery. Another patient developed femoral neck shortening and opted for non-surgical intervention. In cases where complications occurred, reoperation was the chosen endpoint for follow-up. Patients achieved full weight-bearing at an average time of 110.5 ± 10.2 days, with a Harris score of 92.3 ± 4.1, indicating excellent hip function (Table 2). Functional recovery post-surgery was satisfactory, and patients demonstrated normal activities such as standing, walking, running, and squatting (Fig. 6).

**Table 2:** Patient's perioperative related surgical situation and follow-up data

Surgical time (min)	Bleeding volume (ml)	Incision length (cm)	Fluoroscopy frequency (times),	Length of hospital stay (d)	Follow-up time (d)	Complete weight-bearing time (d)	Harris score (points)	Femoral head necrosis	Non healing	Femoral neck shortening
32.6 ± 12.4	55.6 ± 10.5	4.2 ± 1.6	10.5 ± 3.5	8.3 ± 3.8	22.4 ± 2.4	110.5 ± 10.2	92.3 ± 4.1	**1**	**0**	1

## Discussion

Garden IV femoral neck fractures present severe complications, including non-union and ischemic necrosis of the femoral head, arising from complete displacement of the fracture end, instability, and significant disruption of blood supply [6]. Research indicates that heightened joint capsule pressure in femoral neck fractures can impede arterial perfusion and venous return, resulting in sustained ischemia and hypoxia in the femoral head [17]. Femoral head necrosis is mainly caused by collateral circulation disorders, femoral head ischemia, and metabolic abnormalities, closely associated with increased joint capsule pressure. Postoperative concerns, particularly non-union and femoral head necrosis, persist as prominent issues in femoral neck fractures, with an incidence rate of up to 24% [18]. Femoral head necrosis may narrow the hip joint gap, leading to surface collapse and functional impairment, significantly affecting patients' quality of life [19]. Anatomically, femoral neck fractures are classified as fractures in the hip joint capsule [20]. Blood supply to the femoral head and femoral neck mainly originates from branches of the internal and external arteries, traversing the joint capsule exterior, supplying over 70% of the blood to the femoral head. Thus, elevated joint capsule pressure may induce ischemia and necrosis of the femoral head at the fracture end. Rational joint capsule decompression holds potential for enhancing patient prognosis, and reducing the risk of femoral head necrosis may yield positive outcomes [17].

Presently, the predominant clinical fixation approach for femoral neck fractures involves the inverted distribution of hollow screws. However, the internal fixation failure rate using this method ranges from 20 to 48% [21]. Inadequate fixation strength and support are pivotal contributors to internal fixation failures in femoral neck fractures. During complex hip joint activities, the triangular nail placement method lacks effective shear resistance. As the fracture complexity increases, shear resistance gradually diminishes, and insufficient support makes the femoral head susceptible to backward and downward twisting, resulting in deformities. Therefore, ensuring effective stability becomes challenging, potentially leading to femoral neck shortening, affecting hip joint function, walking posture, speed, and causing internal fixation failure of the femoral neck [22].

In recent years, the continuous exploration by both national and international scholars into femoral neck fractures has resulted in advancements in hollow nail treatments. Kauffman's investigation highlights that supplementing the original three screws with an additional parallel screw effectively enhances fixation strength, particularly in cases of comminuted posterior wall fractures [23]. This study also demonstrates that introducing a transverse screw alongside the initial three parallel screws establishes a lower support pier, mitigating displacement of the fracture end to some extent and improving the fixation stability of Garden IV fractures. Biomechanical studies reveal that the incorporation of four cross screws yields a 70% increase in support strength compared to the traditional three parallel screws, providing superior stability in completely displaced fractures [24, 25].

As a result, the author has implemented an innovative hollow nail to augment therapeutic outcomes for Garden IV femoral neck fractures. The femoral neck capsular decompression support hollow screw, devised by this research team, serves the dual role of continuous joint capsular decompression and medial static stabilization support. This inventive approach delivers robust support and fixation for the femoral neck while alleviating pressure on the joint capsule at the fracture end. The study illustrates that this fixation method effectively decompresses pressure in the femoral neck capsule. Postoperative angiography reveals well-developed nutritional blood vessels in the femoral neck and femoral head, ensuring an intact blood supply foundation crucial for femoral neck fracture healing. Moreover, the application of this screw manifests heightened shear resistance and support force, transforming shear force into pressure, thereby increasing compressive strength and offering stable support to maintain precise reduction, anti-inversion, and enhanced weight-bearing capacity. The steeper screw orientation angle aids in resisting entropion to the shaft, minimizing trabecular sagging, facilitating easier sliding of osteoporotic fractures during insertion, and reducing shortening during load-bearing. This design feature prevents severing and ensures enduring fixation strength.

In contrast to prevailing technologies, our team has introduced a groundbreaking femoral neck capsule decompression support screw, colloquially known as the fourth screw. The fourth screw channel is strategically positioned, entering through the greater trochanter of the femur, exiting from the lesser trochanter, nailing into the femoral head, and finally tilting and supporting beneath the femoral neck. This innovative design serves as a comprehensive replacement for traditional bone plates, effectively withstanding shear forces and contributing to the fixation and healing of fractures. Moreover, the decompression support screw in the femoral neck capsule of this utility model is hollow, featuring a side through-hole connected to the screw's front and middle. Placed below the femoral neck, this through-hole precisely aligns inside the joint capsule, allowing fluid in the capsule post-fracture to flow into the hollow part of the screw. It subsequently drains from the tail of the screw to the greater trochanter of the femur, entering soft tissue outside the capsule for absorption and thereby effectively reducing pressure. This screw achieves the objective of avoiding an additional opening in the joint capsule without causing damage to vascular tissue outside the joint capsule. Minimally invasive surgery minimizes damage, facilitating efficient drainage. The decompression support screw showcases innovative and strategic design, featuring a side through-hole in the middle of the screw front. This feature not only provides support for femoral neck fractures, but also facilitates intracapsular decompression with minimal damage and high therapeutic efficacy. The fourth screw channel could potentially be the "golden channel" for treating femoral neck fractures.

Nevertheless, the study is not without limitations: (1) the absence of a control group necessitates additional experiments, including comparative studies with current mainstream treatment methods. (2) The technology imposes high requirements and entails a lengthy learning curve. (3) Stringent demands for intraoperative fluoroscopy further characterize the challenges. (4) Current study lacks biomechanical evidence, with relevant studies currently underway. (5) The included cases are relatively limited, urging an expansion of case studies for comprehensive statistical analysis. The data remain incomplete, necessitating a substantial number of additional case studies. Ongoing efforts involve accumulating more clinical data and case numbers for thorough statistical analysis. (6) The follow-up time remains insufficient, and we are closely monitoring developments.

## Conclusion

After an initial clinical observation, it has been found that the closed reduction femoral neck capsule decompression support hollow screw can offer both postoperative support and capsule decompression for femoral neck fractures. This contributes to enhanced fracture healing and reduces the risk of femoral head necrosis. The approach demonstrates positive therapeutic effects, particularly for Garden IV type femoral neck fractures, warranting promotion and application when indications align. However, further extensive research and exploration are imperative for a comprehensive understanding.

## Data Availability

No datasets were generated or analysed during the current study.

## Abbreviation

CS: Cannulated screws
